# Adrenergic stimulation sensitizes TRPV1 through upregulation of cystathionine *β*-synthetase in a rat model of visceral hypersensitivity

**DOI:** 10.1038/srep16109

**Published:** 2015-11-03

**Authors:** Liyan Zhu, Liting Zhao, Ruobing Qu, Hong-Yan Zhu, Yongmeng Wang, Xinghong Jiang, Guang-Yin Xu

**Affiliations:** 1 Jiangsu Key Laboratory of Translational Research and Therapy for Neuro-Psycho-Diseases, Department of Neurobiology and Physiology, Institute of Neuroscience, Soochow University, Suzhou 215123, P.R. China; 2 Center for Translational Medicine, the Affiliated Zhangjiagang Hospital of Soochow University, Zhangjiagang, 215600, P.R. China

## Abstract

The pathogenesis of pain in irritable bowel syndrome (IBS) is poorly understood and treatment remains difficult. The present study was designed to investigate roles of adrenergic signaling and the endogenous hydrogen sulfide producing enzyme cystathionine *β*-synthetase (CBS) in a previously validated rat model of IBS induced by neonatal colonic inflammation (NCI). Here we showed that NCI-induced visceral hypersensitivity (VH) was significantly attenuated by β_2_ subunit inhibitor but not by β_1_ or β_3_ or α subunit inhibitor. NCI markedly elevated plasma norepinephrine (NE) concentration without alteration in expression of β_2_ subunit receptors in dorsal root ganglion (DRGs) innervating the colon. In addition, NCI markedly enhanced TRPV1 and CBS expression in the colon DRGs. CBS inhibitor AOAA reversed the upregulation of TRPV1 in NCI rats. *In vitro* experiments showed that incubation of DRG cells with NE markedly enhanced expression of TRPV1, which was reversed by application of AOAA. Incubation of DRG cells with the H_2_S donor NaHS greatly enhanced TRPV1 expression. Collectively, these data suggest that activation of adrenergic signaling by NCI sensitizes TRPV1 channel activity, which is likely mediated by upregulation of CBS expression in peripheral sensory neurons, thus contributing to chronic visceral hypersensitivity.

Irritable bowel syndrome (IBS) is defined by recurrent symptoms of visceral pain or discomfort associated with alterations in bowel habits. It remains a common and challenging disorder for clinicians^1^,^2^. The pathophysiology of pain in IBS involves psychological disorder^3^,^4^, altered intestinal motility^5^,^6^ and visceral hypersensitivity^7^,^8^. However, the exact causes of IBS have not been clearly elucidated and effective therapeutics for the primary symptoms have been unavailable. Recent studies in rodents found that early life trauma in the form of neonatal colonic inflammation (NCI) induced visceral hypersensitivity at adult, mimicking the main pathophysiological features of IBS in human^8^,^9^,^10^,^11^. Indeed, early traumatic experiences such as severe diarrhea or life-threatening situations during childhood have been shown to increase the risk of IBS development^12^. The NCI-induced visceral hypersensitivity is distinct from those of inflammatory pain and neuropathic pain in that it produces visceral hyperalgesia without involving inflammatory responses in the gut mucosa and muscle layers in adult^8^,^11^; the latter is characteristic of IBS. Therefore, NCI rats have been used as an animal model to study the mechanisms of IBS.

Alterations in adrenergic signaling have been implicated in the development of visceral hypersensitivity^13^,^14^. It is reported that chronic stress may induce abnormal expressions of brain G proteins, colonic alpha (2A)-adrenoceptors, and norepinephrine reuptake transporter, which may be responsible for the abnormalities of abdominal sensation in IBS^15^. Heterotypic chronic stress can increase sympathetic nervous system activity and induces the release of NE^16^. Once released, NE binds to its receptors. The receptors for NE are a class of G protein-coupled receptors, including α and β subtypes. The α receptors have α_1_ and α_2_ subtypes while the β receptors have β_1_, β_2_ and β_3_ subtypes. The β_1_ and β_2_ adrenergic receptors (ARs) were involved in the adrenergic activation of electrogenic K^+^ secretion in guinea pig distal colonic epithelium^17^, which may play a role in colonic transit. The β_2_ARs located on primary afferent nociceptors are reported to produce a hyperalgesic state in rats^18^. The β_3_ARs, mainly expressed in brown and white adipose tissue, can regulate energy metabolism and thermogenesis^19^. Previous study showed that the blockade of both α_1_/α_2_- and β_1_/β_2_-ARs before the daily application of chronic stress prevented the induction of visceral hypersensitivity in male Wistar rats^11^. However, which subtype of ARs is involved in the induction of visceral hypersensitivity following neonatal colonic inflammation remains unknown.

We have previously reported that the endogenous hydrogen sulfide (H_2_S) producing enzyme cystathionine *β*-synthetase (CBS) was co-localized with transient receptor potential vanilloid 1 (TRPV1) in colon specific DRG neurons, indicative of interaction between these two molecules^20^,^21^. TRPV1 has been shown to play a significant role in both the initiation and the maintenance of visceral hypersensitivity in NCI rat model^11^. However, how these two molecules interact and whether adrenergic activation regulates expression of CBS and TRPV1 remain unknown under NCI conditions. Therefore, we hypothesize that adrenergic signaling is involved in NCI-induced visceral hypersensitivity through sensitization of TRPV1 receptors by CBS-H_2_S signaling. To test this hypothesis, western blotting, patch clamp recordings, calcium imaging and behavioral studies were performed. We demonstrated that NCI led to a significant increase in blood concentration of NE and upregulation of CBS and TRPV1 expression in colon related DRGs. Blockage of CBS suppressed TRPV1 expression and attenuated visceral hypersensitivity. In addition, application of NE enhanced visceral sensitivity and sensitized TRPV1 while inhibition of β_2_ARs attenuated visceral hypersensitivity. Our findings implicate an important role for adrenergic signaling in IBS-like visceral hypersensitivity and identify the β_2_ adrenergic receptors as a potential neurobiological target for the treatment of this symptom.

## Materials and Methods

### Induction of chronic visceral hyperalgesia (CVH)

Experiments were performed on male Sprague-Dawley (SD) rats. Care and handling of these animals were approved by the Institutional Animal Care and Use Committee of the Soochow University and were in accordance with the guidelines of the International Association for the Study of Pain. The CVH was induced by neonatal colonic inflammation (NCI), as described previously^8^,^11^. In brief, ten-day-old pups received an infusion of 0.2 ml of 0.5% acetic acid (AA) solution in saline into the colon 2 cm from the anus. Control rats received an equal volume of normal saline (NS). Experiments were performed in adult rats between 7 and 12 weeks of age. A total of 90 rats were used in the present study.

### Behavioral testing for visceromoter responses

CVH was measured at the age of 6 weeks by grading the behavioral response of rats to colorectal distention (CRD) as described previously^8^,^11^,^22^. Briefly, under mild sedation (1% Brevital, 25 mg/kg, intraperitoneally), CRD was performed by rapidly inflating the balloon to constant pressure using a sphygmomanometer. The balloon was inflated to 20, 40, 60 and 80 mmHg, for 20 s followed by 2 min rest. Behavioral response to CRD was measured by visual observation of the abdominal withdrawal reflex (AWR), and AWR scores were scored either 0 (normal behavior), 1 (slight head movement without abdominal response), 2 (contraction of abdominal muscles), 3 (lifting of abdominal wall) or 4 (body arching and lifting of pelvic structures). To minimize the possible insult from the repetitive distention stimuli of the colon, distension threshold (DT) was measured in this study. DT was the minimal distention pressure to evoke abdominal visceromotor response. It was recorded in mmHg by giving a steady increase in distention pressure by sphygmomanometer. All behavioral tests were performed in a blinded manner.

### Cell labeling

DRG neurons innervating the colon were labeled by injection of 1,1′-dioleyl-3, 3,3′,3-tetramethylindocarbocyanine methanesulfonate (DiI, Invitrogen) into the colon wall^8^. After the injection, rats were returned to their housing and given free access to drinking water and standard food pellets.

### Dissociation of DRG neurons and patch clamp recording

Ten days after DiI injection, NCI rats (7 weeks) and age-mateched control rats were sacrificed by cervical dislocation, followed by decapitation using the methods described previously^23^. DRGs (T_13_-L_2_) were bilaterally dissected out and transferred to an ice-cold, oxygenated fresh dissecting solution. The dissecting solution contains (in mM): 130 NaCl, 5 KCl, 2 KH_2_PO_4_, 6 MgSO_4_, 1.5 CaCl_2_, 10 glucose and 10 HEPES, pH 7.2 (osmolarity = 305 mOsm). The enzymes used for digestion were collagenase D (1.8~2.0 mg/ml; Roche, Indianapolis, Indiana, USA) and trypsin (1.2~1.5 mg/ml; Sigma, St Louis, Missouri, USA). DNase (0.5 mg/ml; Sigma, St Louis, Missouri, USA) was used during repeated trituration to make a single cell suspension. Isolated cells were plated onto acid-cleaned glass coverslips. Under the fluorescence and bright-field microscope (Olympus IX71, Japan), DiI-labeled neurons were identified for the patch-clamp recording experiments. The normal external solution contains (in mM): 130 NaCl, 5 KCl, 2.5 CaCl_2_, 2 KH_2_PO_4_, 1 MgCl_2_, 10 glucose, 10 HEPES, with pH adjusted to 7.2 with NaOH, osmolarity: 295~300 mOsm). The pipette solution contains (in mM): 140 potassium gluconate, 10 NaCl, 5 EGTA, 10 HEPES, 10 glucose and 1 CaCl_2_, with pH = 7.25 adjusted with KOH; osmolarity: 292 mOsm. Capsaicin (CAP) evoked responses were recorded with a HEKA EPC10 patch-clamp amplifier. Data were acquired and stored on a computer for later analysis using FitMaster (HEKA; Germany). Patch clamp recordings were performed at room temperature (~22 °C).

### Western blotting

DRGs (T_13_-L_2_ or L_4_-L_5_) from NCI rats (7 weeks) or age-mateched control rats were dissected out for measuring the expression of CBS, TRPV1 or β2-receptors. Primary antibodies were rabbit anti-CBS, TRPV1 or β2-receptors (1:1000; Alomone Labs, Jerusalem, Israel) and mouse anti-β-actin (1:1000; Chemicon, Temecula, CA). For quantification of protein levels, the photographs were digitalized and analyzed using a scanner (Bio-Rad imaging system Bio-Rad GelDoc XRS + ). All samples were normalized to β-actin as control.

### Measurement of norepinephrine in plasma

Blood samples were collected from the trunk in tubes containing 2.5% sodium citrate and 0.45% citric acid at the time of animal euthanasia by decapitation. The tubes were spun in a refrigerated centrifuge; plasma was quickly aliquoted and stored at −80 °C for assays. Plasma levels of norepinephrine (NE) were measured using radioimmunoassay kits from Abnova (Norepinephrine ELISA Kit), as described previously^24^.

### Quantitative real-time RT-PCR

Total RNA was extracted from DRG samples using Qiagen RNeasy mini kit (Qiagen, Valencia, CA) and 1 μg of total RNA was reverse transcribed using the One-step reverse transcriptase (RT)-PCR kit (Qiagen), according to the manufactures instructions. The expression levels of CBS were quantified by a real-time RT-PCR analysis using SYBR Green I detection kit (Qiagen). PCR reactions were carried out on the ABI PRISM 7900HT Sequence Detection System. Control quantative (Q)-PCR reactions were performed in the absence of cDNA templates. β-actin was used as a housekeeping gene. The primers for CBS were 5′- GAACCAGACGGAGCAAACAG-3′ (forward) and 5′- GGCGAAGGAATCGTCATCA-3′ (reverse), giving a 121 bp amplicon.

### Calcium imaging

DRG neurons were loaded with fura-2 acetoxymethyl ester (2 μM; Invitrogen, Carlsbad, CA) for 30 min at 37 °C in an atmosphere of 5% CO_2_, as described previously^24^. The ratio (R) of fluorescence signal measured at 340 nm, divided by the fluorescence signal measured at 380 nm, is proportional to [Ca^2+^]_i_. Baseline [Ca^2+^]_i_ was determined from the average of five to eight measurements obtained before drug application. The agonists were applied alone, washed out, and then applied again in the continuous presence of NE. Amplitudes of peak [Ca^2+^]_i_ responses were computed as the difference between the peak value and the baseline value.

### Drug application

O-(Carboxymethyl) hydroxylamine hemihydrochloride (AOAA, an inhibitor of CBS), propranolol (Prop, an antagonist of β adrenergic receptor), phentolamine (Phen, an antagonist of α adrenergic receptor), atenolol, SR 59230 A and butoxamine, norepinephrine (NE) and capsaicin (CAP) were purchased from Sigma-Aldrich and were freshly prepared in 0.9% normal saline. AOAA or NE was intraperitoneally injected once daily for consecutive 7 days for molecular expression experiments and for behavioral test.

### Data analysis

All data are expressed as means ± S.E.M. Statistical testing was carried out using a stepwise procedure depending upon the number of groups being compared. Normality was checked for all data before analysis. When only two means were involved in a comparison, a two-tailed t test with unequal variances was used. When more than two means were involved, a one-way analysis of variance or Friedman ANOVA as appropriate, was first carried out to obtain a global test of the null hypothesis. If the global p value for the test of the null hypothesis was <0.05, post hoc comparisons between the different groups using Mann-Whitney test or Dunn’s post hoc test following were performed. A comparison was considered statistically significant when a p value was <0.05.

## Results

### Adrenergic β subunit inhibitor propranolol suppresses VH in NCI rats

**Figure 1:**
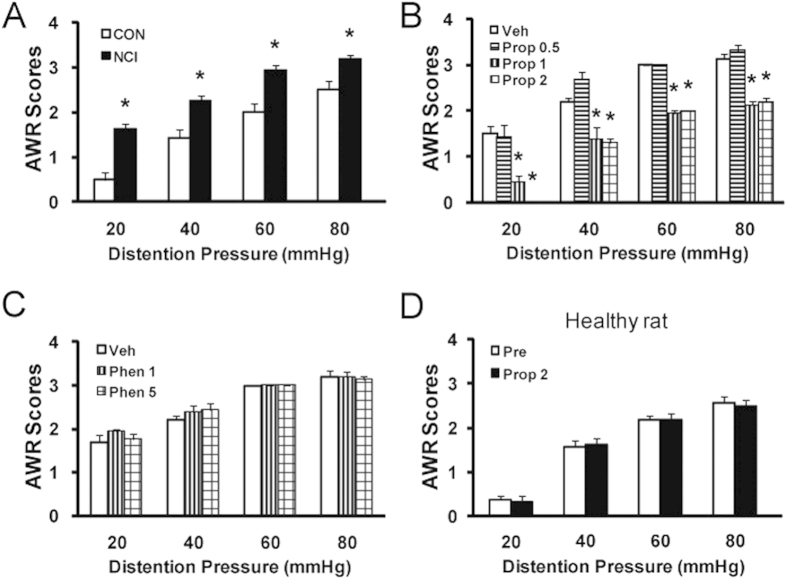
Suppression of visceral hypersensitivity by βAR antagonist. (**A**) Neonatal colonic inflammation (NCI) significantly increased the visceromoter response to colorectal distention (CRD) 6 weeks after neonatal colonic infusion of acetic acid (AA). *p < 0.05, compared with control (CON). (**B**) Systemic administration of nonselective βAR antagonist propranolol (Prop) attenuated NCI-induced visceral hypersensitivity. *p < 0.05, compared with vehicle (Veh). (**C**) αAR antagonist phentolamine (Phen) did not produce any effect in NCI-treated rats. (**D**) Administration of Prop at 2 mg/kg did not produce any effect on AWR scores in age-matched healthy control rats.

Visceral hypersensitivity (VH) was determined by measuring AWR scores in response to colorectal distention (CRD) at 7 weeks of age from normal saline- (CON) or AA-injected (NCI) rats. In an agreement with previous report^8^, AWR scores were significantly higher in NCI rats at 20, 40, 60 and 80 mmHg distention pressures than those in age matched control rats (Fig. 1A, n = 8 for each group, *p < 0.05 vs. CON for the same pressure, Mann-Whitney test following Friedman ANOVA). To determine whether adrenergic signaling is involved in NCI-induced VH, non-selective adrenergic receptor inhibitors, propranolol (Prop) or phentolamine (Phen), was administered intraperitoneally (i.p.). Prop and Phen were used to block β and α adrenergic receptors, respectively. Injection of Prop significantly reduced AWR scores in NCI rats (Fig. 1B, n = 8 for each group, *p < 0.05 vs. NS, Tukey post hoc test following Kruskal-Wallis ANOVA). The optimized dose for Prop was 2 mg/kg body weight in the present study, indicating an analgesic effect of Prop. In contrast, injection of Phen with two different doses failed to alter AWR scores in NCI rats (Fig. 1C), indicating that α adrenergic receptors was not involved in NCI-induced pain processing in the present study. To further confirm the specific effect of Prop, Prop was injected (i.p.) into age-matched healthy control rats. Application of Prop at 2 mg/kg had no significant effects on AWR scores in healthy control rats (Fig. 1D, n = 8 for healthy group). These data suggest that Prop did not act as a non-specific analgesic and that β receptor-mediated adrenergic signaling does not normally participate in the responses to CRD in rats under normal conditions.

### Adrenergic β_2_ subunit inhibitor butoxamine attenuates VH in NCI rats

**Figure 2:**
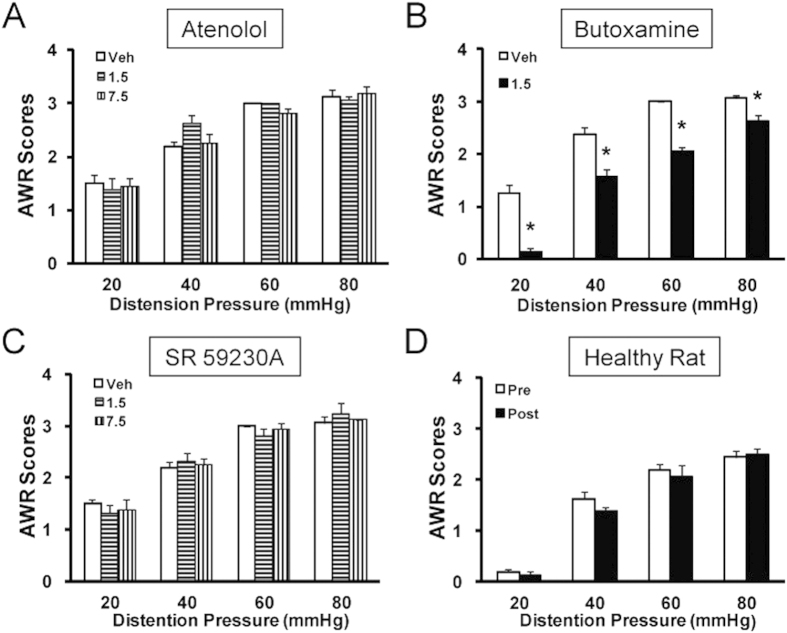
Suppression of visceral hypersensitivity by selective β_2_AR antagonist. (**A**) Administration of selective β_1_AR antagonist atenolol at 1.5 mg or 7.5 mg/kg (i.p.) did not alter AWR scores. (**B**) Administration of selective β_2_AR antagonist butoxamine at a dose of 1.5 mg/kg (i.p.) significantly attenuated AWR scores in NCI rats. *p < 0.05 vs. Veh. (**C**) Administration of selective β_3_AR antagonist SR5923A at doses of 1.5 and 7.5 mg/kg (i.p.) had little effect on AWR scores in NCI rats. (**D**) Administration of butoxamine did not produce any effect on AWR scores in age-matched healthy rats.

To determine which subtype of adrenergic β receptors is involved in NCI-induced VH, three selective subtype antagonists, atenolol, butoxamine and SR 59230 A, were used to block β_1_, β_2_ and β_3_ receptors, respectively. The concentrations used for these inhibitors were referenced by our previous studies^25^. Butoxamine at the dose of 1.5 mg/kg body weight (i.p.) significantly attenuated the AWR scores in NCI rats (Fig. 2B, n = 8). However, atenolol at doses of 1.5 and 7.5 mg/kg (Fig. 2A, n = 8) or SR 59230 A at doses of 1.5 and 7.5 mg/kg (Fig. 2C, n = 8) did not produce any effect on AWR scores in NCI rats. To further confirm the specific effect of butoxamine, butoxamine was injected into age-matched healthy control rats. Application of butoxamine at dose of 1.5 mg/kg (i.p.) had no significant effects on AWR scores in healthy control rats (Fig. 2D, n = 8 for healthy group). Collectively, these data indicate that VH is likely mediated by β_2_-adrenergic receptors in NCI rats.

### NCI increases norepinephrine concentration in blood plasma without alteration in expression of β_2_ adrenergic receptors in DRGs

**Figure 3:**
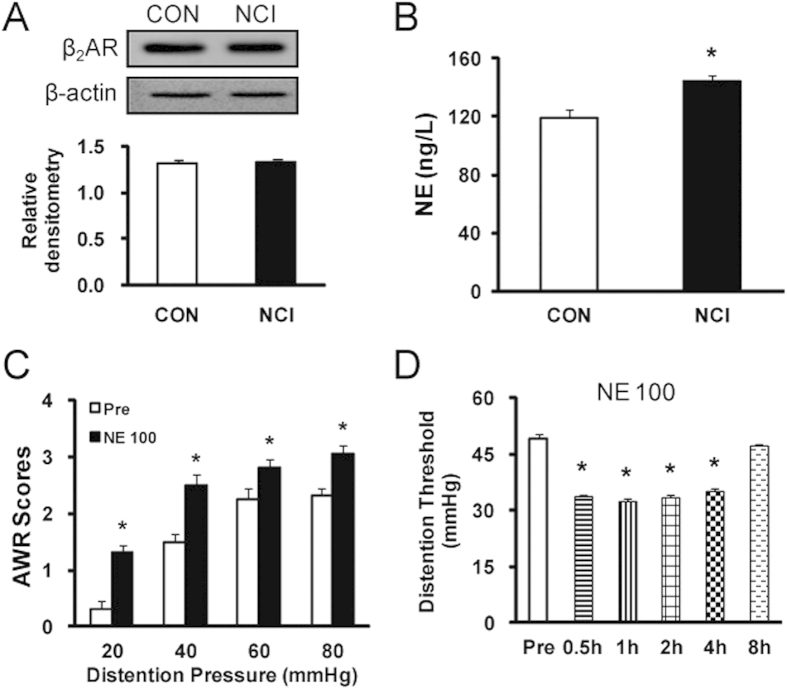
Increase in plasma concentration of norepinephrine (NE) after NCI. (**A**) NCI did not alter expression of β_2_ARs in colon DRGs. (**B**) NCI markedly enhanced concentration of NE in blood plasma. *p < 0.05 vs. control (CON). (**C**) Application of NE (100 μg/1ml, i.p.) enhanced AWR scores in healthy rats. *p < 0.05 vs. baseline (Pre). (**D**) Time course of effect of NE at 100 μg/ml. Application of NE reduced distention threshold, starting at 0.5h and maintaining for at least 4h within our observation time period. *p < 0.05, compared with Pre.

We next determine whether NCI enhanced the expression of β_2_ receptors in colon DRGs. Surprisingly, expression of β_2_ receptors was not significantly altered after NCI treatment (Fig. 3A). Since NE is one of most prominent mediators of stress response^25^,^26^, we assayed the plasma levels of NE to investigate whether NE is a candidate to induce persistent colonic hyperactivity in response to NCI. The blood plasma concentration of NE was 119.51 ± 5.31 and 143.89 ± 4.02 in control and NCI rats, respectively. NCI slightly but significantly increased NE concentration when compared with that of age-matched controls (Fig. 3B, *p < 0.05, n = 14 for each group). To further confirm the role of NE on AWR scores, NE at 100 μg/1 ml for each rat was injected (i.p.) into healthy control rats once daily for consecutive 7 days. Application of NE markedly increased AWR scores (Fig. 3C, n = 8, *p < 0.05), and this effect lasted for ~4 hours after (Fig. 3D, n = 8, *p < 0.05). These data indicate a proalgesic role for NE.

### NE application sensitizes TRPV1

**Figure 4:**
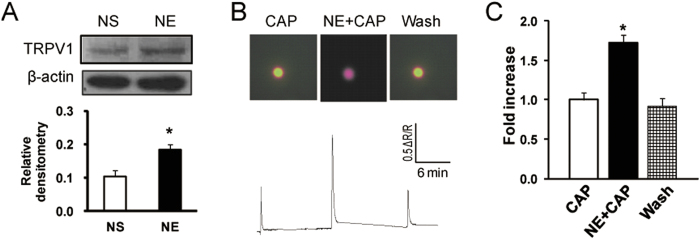
Potentiation of capsaicin-induced responses in DRG neurons innervating the colon. (**A**) Application of NE (100 μg/1ml, i.p.) once daily for 7 consecutive days significantly increased TRPV1 expression in T13-L2 DRGs when compared NS treated-rats. (**B**) An example from a cell showing responses to capsaicin (CAP, 1 μM) before and after incubation of NE (10 μM) for 5 minutes. (**C**) Bar graph showing a potentiation of CAP-induced Ca^2+^ transients in colon specific DRG neurons by NE. *p < 0.05, compared with CAP.

To further confirm the effects of NE on visceral hypersensitivity, we examined the expression and function of TRPV1 in DRGs after 7 consecutive day application of NE (100 μg/ml) in healthy control rats. Application of NE significantly enhanced expression of TRPV1 in colon DRGs. The relative densitometry of TRPV1 was 0.10 ± 0.02 (n = 4) and 0.18 ± 0.02 (n = 4) for NS- and NE-treated group, respectively (Fig. 4A, *p < 0.05). Application of capsaicin (CAP, 1 μM) produced a transient increase in intracellular Ca^2+^concentration in ~80% of neurons tested (Fig. 4B). The ΔR/R was 0.75 ± 0.09 (n = 5) before NE application. Five minutes after NE application, ΔR/R was 1.29 ± 0.10 (n = 5). The increase in amplitude was statistically significant (Fig. 4C, n = 5 cells, *p < 0.05). The CAP-induced intracellular calcium mobilization was returned to baseline after removal of NE (Wash). These data indicate the involvement of adrenergic signaling in sensitization of TRPV1.

### NCI sensitizes TRPV1 channels through adrenergic signaling

**Figure 5:**
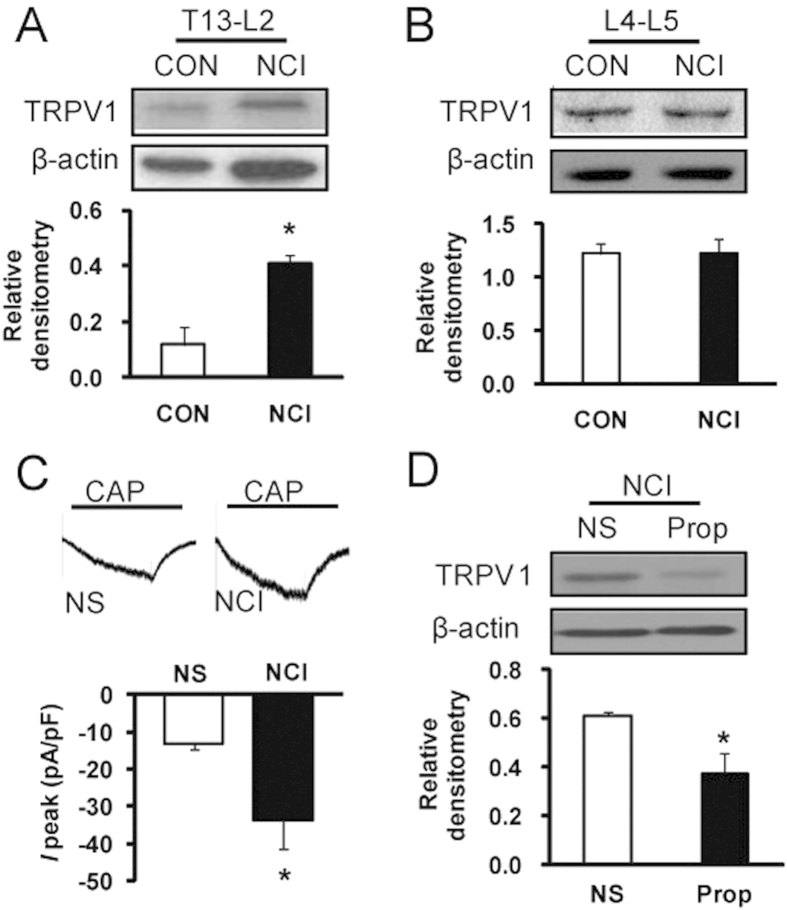
Sensitization of TRPV1 in NCI rats. (**A**) NCI markedly enhanced expression of TRPV1 in colon DRGs (T13, L1 and L2) when compared with control (CON). *p < 0.05 vs. CON. (**B**) NCI did not alter TRPV1 expression in lumbar DRGs (L4 and L5). (**C**) Capsaicin (CAP, 1 μM)-induced currents were significantly greater in NCI rats than in NS rats. *p < 0.05. (**D**) Application of Prop (2 mg/kg, i.p.) once daily for 7 consecutive days significantly reduced TRPV1 expression in colon DRGs. *p < 0.05 vs. NS.

We next determined whether NCI enhanced TRPV1 expression and function. In an agreement with previous report^11^, NCI greatly enhanced TRPV1 expression in T13-L2 DRGs when compared with age-matched controls (Fig. 5A, *p < 0.05). The relative densitometry of TRPV1 was 0.12 ± 0.06 (n = 3) and 0.41 ± 0.03 (n = 3) for CON and NCI rats, respectively. To confirm the specificity of TRPV1 expression in colon DRGs, expression of TRPV1 in lumbar L4-5 DRGs was studied and was used as controls since L4-5 DRGs are mainly innervating the hindlimbs. The relative densitometry of TRPV1 was 1.22 ± 0.10 (n = 3) and 1.22 ± 0.15 (n = 3) for CON and NCI rats, respectively. NCI did not significantly alter expression of TRPV1 in L4-5 DRGs (Fig. 5B). We then determined whether TRPV1 function was enhanced after NCI. Capsaicin (CAP, 1 μM) was used to record CAP-evoked current at holding membrane potential of −60 mV in DiI labeled colon specific DRG neurons. The average current density was −12.98 ± 1.73 pA/pF (n = 10 cells) and −33.56 ± 2.91 pA/pF (n = 7 cells) from CON and NCI rats, respectively. Compared with control, NCI significantly enhanced CAP-evoked peak current density (Fig. 5C, *p < 0.05). We then investigated the interaction of NE signaling and TRPV1 expression. Treatment with selective β–receptor antagonist Prop (2 mg/kg body weight, i.p.) once daily for consecutive 7 days markedly suppressed expression of TRPV1 in DRGs from NCI rats (Fig. 5D, *p < 0.05). The relative densitometry of TRPV1 was 0.12 ± 0.06 (n = 3) and 0.30 ± 0.01 (n = 3) for NS- and Prop-treated rats, respectively.

### CBS inhibitor suppresses TRPV1 upregulation in NCI rats

**Figure 6:**
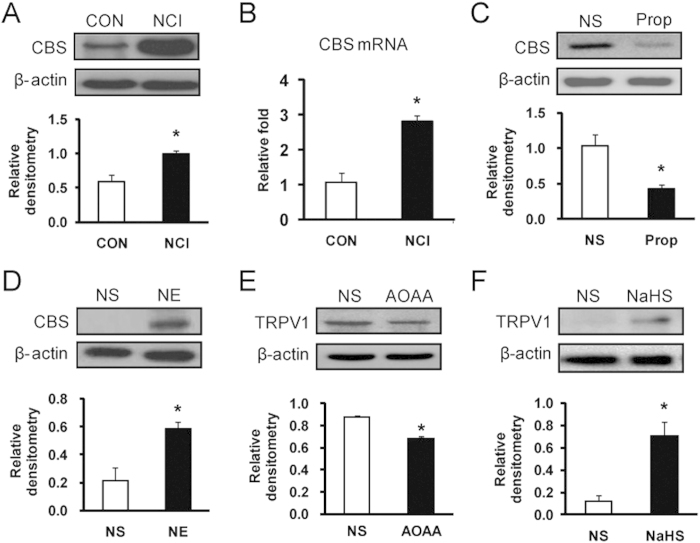
Upregulation of CBS and TRPV1 expression in NCI rats. (**A**) NCI markedly enhanced expression of CBS at protein levels in colon DRGs. *p < 0.05, compared with CON. (**B**) NCI significantly enhanced expression of CBS at mRNA levels in colon DRGs. *p < 0.05, compared with CON. (**C**) Application of Prop (2 mg/kg, i.p.) once daily for 7 consecutive days significantly reduced CBS expression in NCI rats when compared with NS rats. *p < 0.05. (**D**) Application of NE (100 μg/1 ml, i.p.) once daily for 7 consecutive days significantly increased CBS expression in colon related DRGs when compared with control (NS). *p < 0.05. (**E**) Application of AOAA (10 mg/kg, i.p.) once daily for 7 consecutive days significantly reduced TRPV1 expression in colon DRGs. *p < 0.05, compared with NS. (**F**) NaHS (100 μM) incubation with DRG cell cultures markedly enhanced expression of TRPV1 (*p < 0.05, compared with NS).

We then determined whether the endogenous hydrogen sulfide (H_2_S) producing enzyme CBS is involved in the NCI-induced TRPV1 upregulation. In a line with previous report^8^, NCI dramatically enhanced CBS expression in T13-L2 DRGs both at protein (Fig. 6A, *p < 0.05, n = 3 for each group) and mRNA level (Fig. 6B, *p < 0.05, n = 3 for each group). Treatment with Prop for consecutive 7 days significantly suppressed expression of CBS in NCI rats (Fig. 6C, *p < 0.05). The relative densitometry of CBS was 1.03 ± 0.15 (n = 3) and 0.42 ± 0.05 (n = 3) for NS- and Prop-treated rats, respectively. In contrast, treatment with NE (100 μg/1 ml) for consecutive 7 days greatly enhanced expression of CBS in healthy controls (Fig. 6D, *p < 0.05). The relative densitometry of CBS was 0.21 ± 0.09 (n = 4) and 0.58 ± 0.05 (n = 4) for NS- and NE-treated rats, respectively. To confirm the role of CBS in modulation of TRPV1 expression, AOAA, an inhibitor of CBS, was used in the present study. Treatment with AOAA (10 mg/kg body weight, i.p.) for consecutive 7 days greatly suppressed expression of TRPV1 in NCI rats (Fig. 6E, *p < 0.05). The relative densitometry of TRPV1 was 0.87 ± 0.02 (n = 3) and 0.68 ± 0.02 (n = 3) for NS and AOAA group, respectively. To further confirmed the effect of H_2_S on TRPV1 expression, *in vitro* studies of cultured DRG neurons with NaHS was performed. NaHS, a donor for H_2_S, was to mimic the production of H_2_S for CBS. Addition of NaHS at 100 μM for 5 hours significantly increased TRPV1 expression when compared with NS treatment (Fig. 6F, *p < 0.05). The relative densitometry of TRPV1 was 0.12 ± 0.05 (n = 3) and 0.70 ± 0.12 (n = 3) for NS- and NaHS-treated cells, respectively. These data indicate an important role for CBS-H_2_S signaling in sensitization of TRPV1 channels.

## Discussion

We demonstrated here that adrenergic signaling plays an important role in neonatal colonic inflammation-induced adult visceral hypersensitivity. The present findings are significant because they provide evidence to support the hypothesis that adrenergic activation plays a crucial role in functional visceral pain such as IBS, which is pain occurring in the absence of overt tissue inflammation or damage of the colon. To prove this, we used a previously validated visceral hypersensitivity model of IBS^11^,^27^, which was established by colonic irritation with diluted acetic acid at the neonatal age. This approach did not induce robotic inflammation or damage of the colon but produced visceral hypersensitivity at adult. Adrenergic signaling has been reported to participate in the inflammatory and neuropathic pain states^24^,^28^. Here we demonstrated that application of propranolol resulted in a significant analgesic effect in IBS rats. Our results suggested that this antinociceptive effect is specific rather than a toxic or non-specific effect since propranolol did not produce any effect on healthy control rats. Therefore, our studies add NE and β_2_ARs to the list of key nociceptive molecules that involve in visceral hypersensitivity of functional gastrointestinal disorders.

The most prominent finding is that NCI elevated the NE concentration in blood plasma without alteration of β_2_AR expression (Fig. 3A,B). NE is one the most prominent mediators of stress responses^29^,^30^. It has been reported to be involved in many pathophysiological processes such as inflammation and pain^31^,^32^. The present study is in a line with previous reports that adrenergic signaling plays a role in a chronic stress model^13^,^25^. Application of NE induced visceral hypersensitivity (Fig. 3C,D), upregulated the expression of CBS (Fig. 6D) and TRPV1 (Fig. 4A), and enhanced the capsaicin-induced intracellular calcium mobilization (Fig. 4B,C) of healthy rats, indicating that NE mimics the effects of neonatal colonic infusion of acetic acid. The mechanism underlying the elevation of NE level is not clear. Several studies suggest that the hypothalamic-pituitary-adrenal axis, the sympathetic system originating in the locus coeruleus and the downregulation of NE reuptake transporters are responsible for the elevated NE level^13^,^15^,^31^. Although the detailed mechanisms for an increase in NE levels have yet to be investigated, our findings indicate that NE might be the major contributor to visceral hypersensitivity in adult rats with NCI, further supporting that NE might be a risky factor for chronic visceral pain in patients with IBS^15^,^25^.

Another important finding is that CBS inhibitor blocked upregulation of TRPV1 expression in NCI rats. In agreement with previous studies, NCI significantly enhanced the expression of CBS^20^ and TRPV1^11^ in colon DRGs. However, mechanisms by which NE upregulates TRPV1 expression remain largely unknown. NE elevated in the blood plasma could increase expression of nerve growth factor in the colon wall, thus sensitizing primary afferents^13^. In the present study, we provided evidence to demonstrate for the first time that activation of CBS bridges the gap between adrenergic activation and TRPV1 expression. This was supported by the *in vitro* and *in vivo* results. Application of H_2_S donor NaHS increased expression of TRPV1 (Fig. 6F) while inhibition of CBS by AOAA blocked upregulation of TRPV1 in the DRGs of adult rats with NCI (Fig. 6E). Previous studies suggest that CBS-H_2_S signaling is involved in inflammatory and neuropathic pain^33^,^34^, and in gastric hypersensitivity in rats with diabetic gastroparesis^35^. In the present study, we provided novel evidence to show that inhibition of adrenergic signaling suppressed the upregulation of CBS and TRPV1, indicating the involvement of CBS and TRPV1 in mediating the NCI-induced visceral hypersensitivity. Together with previous report that application of AOAA attenuates the visceral hypersensitivity in NCI rat^21^, the present study adds CBS-H_2_S to the list of key nociceptive genes that involve in functional visceral hypersensitivity. How CBS-H_2_S mediates TRPV1 sensitization remains largely unknown. The possible mechanisms include the sulfuration of reactive cysteine residues in TRPV1^36^.

In summary, these data provide evidence that NE elevated in blood plasma activates β_2_-adrenergic receptors to enhance the expression of CBS and TRPV1 of colon DRGs, thus producing visceral hypersensitivity. Blockade of β_2_ARs attenuated the visceral hypersensitivity to colorectal distension after NCI. These findings suggest that adrenergic pathway might be a potential target for novel agents for the treatment of visceral pain in patients with IBS.

## Additional Information

**How to cite this article**: Zhu, L. *et al.* Adrenergic stimulation sensitizes TRPV1 through upregulation of cystathionine *β*-synthetase in a rat model of visceral hypersensitivity. *Sci. Rep.* **5**, 16109; doi: 10.1038/srep16109 (2015).
